# From static risk to dynamic disease monitoring: the role of MRD and immune profiling in multiple myeloma

**DOI:** 10.3389/fimmu.2026.1934257

**Published:** 2026-08-20

**Authors:** Maria Eugenia Alvaro, Enrica Antonia Martino, Santino Caserta, Antonella Bruzzese, Nicola Amodio, Eugenio Lucia, Virginia Olivito, Caterina Labanca, Francesco Mendicino, Fortunato Morabito, Ernesto Vigna, Massimo Gentile

**Affiliations:** 1 Hematology Unit, Department of Onco-Hematology, Azienda Ospedaliera (AO) of Cosenza, Cosenza, Italy; 2 Department of Experimental and Clinical Medicine, University of Catanzaro, Catanzaro, Italy; 3 Associazione Italiana contro le Leucemie - Linfomi e Mieloma (AIL) Sezione di Cosenza, Cosenza, Italy; 4 Department of Pharmacy, Health and Nutritional Science, University of Calabria, Rende, Italy

**Keywords:** bispecific antibodies, CAR T cells, dynamic risk assessment, immune profiling, immune reconstitution, liquid biopsy, measurable residual disease, minimal residual disease

## Abstract

The therapeutic landscape of multiple myeloma (MM) has undergone a profound transformation, with highly effective combination regimens and immune-based therapies enabling unprecedented rates of deep and durable responses. As a result, conventional baseline risk stratification alone is increasingly insufficient to explain the heterogeneity of clinical outcomes or to guide treatment throughout the disease course. This evolving paradigm has shifted attention toward dynamic, response-adapted disease monitoring centered on measurable residual disease (MRD) and the biological interaction between residual tumor cells and the host immune system. Bone marrow-based next-generation flow cytometry and next-generation sequencing currently represent the most extensively validated approaches for MRD assessment, while functional imaging, mass spectrometry, circulating tumor DNA, and other minimally invasive technologies are expanding the ability to monitor spatially heterogeneous disease and longitudinal clonal evolution. Although sustained MRD negativity has emerged as one of the most powerful prognostic biomarkers in MM, its clinical significance is influenced by multiple factors, including timing of assessment, sensitivity, durability of response, baseline disease biology, imaging findings, and the quality of immune reconstitution. Increasing evidence indicates that relapse following MRD negativity reflects not only residual tumor burden below the limits of detection but also a dynamic biological process driven by clonal evolution, microenvironmental protection, immune escape, and therapeutic selection pressure. Immune profiling provides complementary information by characterizing immune competence, including T-cell and natural killer-cell function, immune reconstitution after therapy, regulatory and myeloid immunosuppressive networks, and the immune fitness required for effective T-cell redirection and the capacity to sustain effective antitumor immune surveillance. Integrating longitudinal MRD kinetics with immune biomarkers has the potential to improve risk discrimination, identify biologically discordant disease states, and support rational strategies for treatment intensification, de-escalation, or discontinuation within prospective clinical trials. In the era of anti-CD38 antibodies, CAR T-cell therapy, bispecific antibodies, and emerging multi-antigen immunotherapies, disease monitoring is evolving beyond the assessment of tumor burden alone. Future of MM management will likely rely on multidimensional monitoring frameworks that integrate MRD, immune competence, spatial disease assessment, circulating biomarkers, and computational risk modeling to enable truly personalized, biology-driven patient care.

## 1 Introduction

Multiple myeloma (MM) is a biologically and clinically heterogeneous plasma-cell neoplasm in which clinical outcomes are determined by the complex interplay between tumor genetics, disease burden, bone marrow microenvironment, and host immunity. Over the past two decades, prognostic assessment has relied primarily on baseline staging systems, including the International Staging System (ISS), the Revised ISS (R-ISS), and, more recently, the Second Revision ISS (R2-ISS), which integrate biochemical markers with high-risk cytogenetic abnormalities to classify patients into clinically meaningful risk categories (1–3). These models remain fundamental for patient counseling, clinical trial stratification, and therapeutic decision-making. However, they were developed in an era in which complete responses represented the principal therapeutic endpoint and before the widespread availability of highly effective quadruplet regimens, anti-CD38 monoclonal antibodies, cellular immunotherapies, and sensitive technologies capable of detecting residual disease beyond conventional response criteria (4).

The therapeutic landscape of MM has changed profoundly. The introduction of proteasome inhibitors, immunomodulatory drugs, anti-CD38 monoclonal antibodies, autologous stem-cell transplantation (ASCT), chimeric antigen receptor (CAR) T-cell therapies, and bispecific antibodies has dramatically increased the frequency of deep and durable responses, with a growing proportion of patients achieving disease levels undetectable by conventional serum and urine assessments (5). Consequently, the central clinical question is no longer limited to the biological risk assigned at diagnosis, but increasingly concerns how residual disease evolves during therapy and how effectively the host immune system can maintain long-term disease control. Reflecting this paradigm shift, the International Myeloma Working Group (IMWG) incorporated measurable residual disease (MRD) assessment, with or without imaging-confirmed absence of extramedullary disease, into contemporary response criteria, recognizing MRD negativity as the deepest currently measurable level of treatment response (6, 7).

MRD has subsequently emerged as one of the most robust prognostic biomarkers in MM. Large meta-analyses have consistently demonstrated that MRD negativity is associated with significantly prolonged progression-free survival (PFS) and overall survival (OS) across newly diagnosed and relapsed or refractory disease, regardless of transplant eligibility or treatment platform (8). However, the interpretation of MRD has become increasingly nuanced. A single negative marrow assessment should not be equated with disease eradication. Relapse despite MRD negativity is now a well-recognized clinical phenomenon, highlighting the biological limitations of considering residual disease solely as a quantitative entity. Residual myeloma cells may survive below current detection thresholds, localize within unsampled marrow sites or extramedullary compartments, or persist through mechanisms of clonal evolution, phenotypic plasticity, metabolic adaptation, microenvironmental protection, and immune escape (9, 10). These observations suggest that MRD represents not only the amount of residual disease but also a biological state whose clinical significance depends on the characteristics of both the tumor and its surrounding ecosystem.

In parallel, contemporary MM therapy increasingly relies on mechanisms that directly engage the immune system. Anti-CD38 antibodies reshape both innate and adaptive immunity, whereas CAR T cells and bispecific antibodies depend on effective T-cell activation, expansion, and persistence to achieve durable responses (11). Consequently, patients with apparently identical MRD status may experience markedly different clinical outcomes depending on the degree of immune reconstitution, T-cell exhaustion, natural killer (NK)-cell function, regulatory immune populations, and the overall balance between immune surveillance and immune suppression (12). MRD therefore provides a highly sensitive measure of residual tumor burden but does not fully capture the biological capacity of the host to eliminate or restrain the remaining malignant clone.

These advances have progressively shifted the focus of prognostication from static baseline risk assessment toward dynamic disease monitoring. Rather than relying exclusively on variables measured at diagnosis, contemporary risk assessment increasingly incorporates longitudinal information derived from MRD kinetics, functional imaging, liquid biopsy technologies, immune profiling, and computational modeling. Together, these complementary approaches provide a more comprehensive representation of disease biology by integrating tumor burden, spatial heterogeneity, immune competence, and treatment-induced evolution.

In this review, we discuss the transition from conventional baseline risk stratification to dynamic, response-adapted monitoring in MM. We summarize current methodologies for MRD assessment, examine their clinical applications and limitations, review the emerging role of immune profiling and immune reconstitution, and explore how the integration of these complementary domains may support increasingly personalized therapeutic strategies in the era of immunotherapy.

Throughout this review, the abbreviation MRD refers to measurable residual disease, defined as residual myeloma detectable by highly sensitive molecular, immunophenotypic, imaging-based, or blood-based methods beyond conventional response criteria. We acknowledge that the term “minimal residual disease” has historically been widely used in MM and remains present in several consensus documents, clinical trials, regulatory texts, and article titles. However, “measurable residual disease” more accurately reflects the operational nature of contemporary assays, which detect disease only to defined limits of detection and do not establish complete disease eradication. For consistency, we therefore use “measurable residual disease” throughout the manuscript, except when referring to historical terminology or to the exact title or wording of cited sources.

Recent reviews have comprehensively summarized MRD platforms, MRD-adapted treatment approaches, immune reconstitution and emerging monitoring technologies in MM (8–10, 12). Rather than providing another method-by-method description of MRD assessment, this review critically examines how measures of tumor-burden, immune competence, spatial disease distribution, and longitudinal biomarkers may be integrated into a biologically coherent monitoring framework. In contrast to reviews focused predominantly on assay performance or MRD-guided therapy, we distinguish established clinical applications from investigational translational strategies. We t therefore position MRD as a validated prognostic biomarker and trial-stratification tool, while emphasizing that immune profiling—and the integration of MRD and immune parameters for therapeutic decision-making—remain incompletely validated. At present, these approaches should inform risk interpretation, clinical trial design, and hypothesis generation rather than routine therapeutic algorithms.

## 2 From static baseline risk to dynamic risk assessment

Baseline staging remains an essential component of MM management. The ISS, the R-ISS, and the recently introduced R2-ISS have progressively refined prognostic assessment by integrating tumor burden, biochemical parameters, and high-risk cytogenetic abnormalities into clinically meaningful risk categories. ISS incorporates serum albumin and beta-2 microglobulin concentrations to estimate disease burden, whereas R-ISS further integrates lactate dehydrogenase levels and specific high-risk cytogenetic abnormalities. More recently, R2-ISS has refined this framework by incorporating additional biological variables, including 1q gain/amplification, and assigning weighted risk components to improve prognostic discrimination (1–3).

Nevertheless, these systems are inherently static. They summarize disease at diagnosis but cannot account for subclonal selection, treatment-induced immune remodeling, emergence of extramedullary disease, or the kinetics and durability of response.

**Figure 1:**
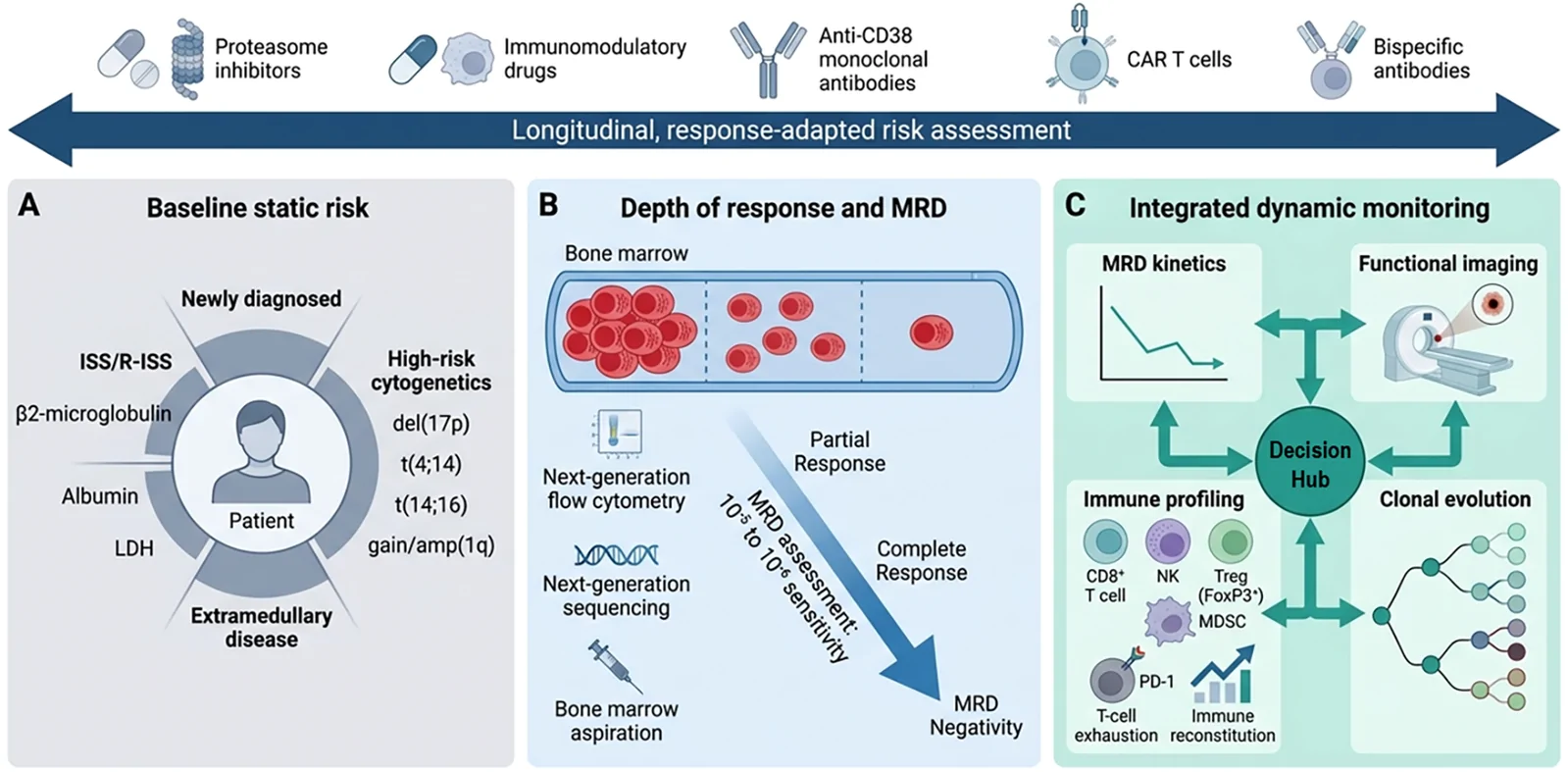
From static risk stratification to integrated dynamic disease monitoring in multiple myeloma. Traditional prognostic models in multiple myeloma rely on baseline clinical, biochemical, and cytogenetic features assessed at diagnosis **(A)**. With the advent of more effective therapies, response depth evaluation, particularly through measurable residual disease (MRD), has become central to outcome prediction **(B)**. Modern disease monitoring integrates MRD kinetics, immune profiling, functional imaging, and biological evolution under therapeutic pressure **(C)**. This multidimensional approach supports response-adapted risk reassessment and provides a conceptual basis for personalized treatment strategies in the era of immunotherapy. CAR T cells, Chimeric Antigen Receptor T; CD38, Cluster of Differentiation 38; ISS/R-ISS, International Staging System/Revised International Staging System; LDH, Lactate Dehydrogenase; MRD, Minimal Residual Disease; NK, Natural Killer; Treg, Regulatory T cell; FoxP3, Forkhead box P3; MDSC, Myeloid-Derived Suppressor Cell; PD-1, Programmed Cell Death Protein 1.

The clinical relevance of this limitation is increasingly evident in contemporary practice. Patients with comparable baseline cytogenetic profiles may experience markedly different outcomes depending on the depth and durability of treatment response, whereas individuals initially classified as standard risk may subsequently develop functionally high-risk disease characterized by persistent MRD, early relapse, or treatment-resistant disease evolution. Conversely, the widespread adoption of intensified approaches, including anti-CD38-containing quadruplet regimens, has improved response depth and may partially mitigate the adverse prognostic impact associated with selected baseline high-risk features (13). These observations indicate that prognosis in MM should no longer be considered a fixed characteristic established at diagnosis, but rather a continuously evolving process that is progressively refined by treatment response, MRD kinetics, imaging findings, and emerging biological biomarkers (14). This transition from static baseline classification toward longitudinal, response-adapted monitoring represents a fundamental change in the way disease risk is conceptualized in MM. **Figure 1** illustrates the evolution of prognostic assessment in multiple myeloma from static baseline risk stratification toward integrated dynamic disease monitoring. By combining MRD kinetics with immune profiling, functional imaging, and disease evolution under therapeutic pressure, this multidimensional framework supports continuous risk reassessment and provides the basis for personalized, response-adapted management.

Dynamic risk assessment therefore requires interpretation across two complementary dimensions. The first is quantitative, addressing whether the malignant plasma-cell compartment has been reduced below the detection threshold of available assays. The second is qualitative, addressing whether the residual disease ecosystem remains capable of persistence, immune escape, and subsequent expansion. MRD primarily informs the quantitative dimension by measuring detectable malignant residual tumor burden, whereas immune profiling provides insight into the biological environment that determines whether residual clones remain controlled or regain proliferative capacity. This distinction has important clinical implications because identical MRD results may represent biologically different states. A patient with sustained MRD negativity accompanied by immune recovery, preserved T-cell receptor diversity, functional natural killer-cell activity, and effective immune surveillance may have a fundamentally different disease trajectory compared with a patient who achieves the same MRD status but exhibits persistent immune dysfunction, hypogammaglobulinemia, T-cell exhaustion, or imaging evidence of residual focal disease (15, 16).

Thus, MRD should increasingly be interpreted not as an isolated endpoint but as one component of a broader biological framework integrating residual tumor burden, immune competence, spatial disease distribution, and longitudinal disease kinetics. This shift from static risk assignment toward dynamic disease monitoring provides the foundation for a more precise and adaptive approach to MM management, particularly in the era of immune-based therapies.

## 3 MRD assessment in multiple myeloma: validated platforms and emerging technologies

MRD assessment in MM has progressively transformed from a highly sensitive measurement of residual tumor burden into a broader strategy for evaluating disease depth, spatial distribution, and biological evolution during treatment. Historically, MRD evaluation has been centered on bone marrow, where residual malignant plasma cells are detected by immunophenotypic or molecular approaches. Among available methods, next-generation flow cytometry (NGF) and next-generation sequencing (NGS) represents the most extensively validated platforms and are incorporated in IMWG response criteria (7). NGF identifies aberrant plasma cells through standardized antibody panels and multidimensional immunophenotypic differences between normal and malignant plasma cell populations. The EuroFlow consortium has substantially improved analytical reproducibility by harmonizing sample preparation, antibody combinations, acquisition requirements, and computational strategies, allowing highly sensitive detection of residual disease across different laboratories. NGS-based MRD assessment relies on patient-specific immunoglobulin gene rearrangements identified at diagnosis and enables molecular tracking of residual clones with sensitivity levels approaching 10^-6 when adequate baseline material and a trackable clonotype are available (17–19).

The major advantages of NGF include broad applicability, rapid turnaround time, and the simultaneous evaluation of malignant plasma cells and immune-cell populations within the same specimen. Conversely, its accuracy depends on sample quality, adequate marrow representation, avoidance of hemodilution, and implementation of standardized analytical procedures. NGS provides high sensitive and reproducible molecular tracking but requires successful identification of a baseline clonotype identification and may be challenging when diagnostic samples are unavailable or insufficient for sequencing. Despite their analytical power, both approaches share an important biological limitation: a single iliac crest aspirate represent only one anatomical compartment and may not fully reflect the overall burden of a spatially heterogeneous disease. This limitation is particularly relevant in patients with patchy marrow involvement, focal lesions, paramedullary extension, or extramedullary disease (19, 20).

For this reason, imaging-based approaches provide an essential complementary dimension by addressing the spatial limitations of marrow-based MRD. Fluorodeoxyglucose positron emission tomography/computed tomography (FDG PET/CT) is currently the most widely used functional imaging modality for response assessment and can identify metabolically active lesions outside the sampled marrow compartment. Emerging approaches, including dual-tracer PET strategies, aim to further improve the detection of biologically active residual disease and characterize disease heterogeneity (21). Importantly, imaging should not be considered an alternative to marrow MRD but rather a complementary strategy that identifies residual disease outside the sampled compartment. In a prospective real-world study of 118 newly diagnosed patients, NGF at a sensitivity threshold of 10^-5 independently predicted inferior PFS and OS, whereas ^11C-acetate PET detected extramedullary metabolic activity in selected patients who were NGF-negative, highlighting the complementary nature of cellular and imaging-based assessments (22).

The need for repeated and minimally invasive disease monitoring has stimulated the development of blood-based approaches. Mass spectrometry has improved the sensitivity of monoclonal proteins beyond conventional electrophoresis and immunofixation by enabling highly specific tracking of patient-derived monoclonal proteins (23). The EasyM approach, which monitors patient-specific clonotypic M-protein peptides, analyzed 447 sequential serum samples from 56 patients and demonstrated high sensitivity compared with conventional immunofixation and cellular MRD methods. In multivariate analysis, the best MS-MRD status was independently associated with both PFS and OS, supporting its potential role as a longitudinal biomarker of disease control (24). Circulating tumor DNA (ctDNA) represents another promising approach for systemic and minimally invasive disease monitoring. By sampling peripheral blood, ctDNA may provide information from multiple anatomical sites and potentially capture clonal evolution beyond the limitations of a single marrow aspirate. In a 2026 study using high-sensitivity tumor-informed NGS in peripheral blood samples from patients enrolled in PETHEMA/GEM trials, ctDNA detection was feasible in most patients, showed high specificity for relapse prediction, and was associated with increased risk of progression and/or death (25). However, the clinical application of ctDNA remains limited by variable shedding patterns, particularly in patients with low-volume disease or predominantly marrow-restricted involvement, and requires further prospective validation.

MRD assessment of autologous stem-cell grafts has also gained renewed interest as an additional biologic marker. Detection of clonal plasma cells in peripheral blood stem-cell collections using flow cytometry, NGF, and NGS may reflect tumor contamination before ASCT, and provide additional prognostic information. However, graft contamination should be interpreted as a risk marker rather than as the primary determinant of relapse, whose biology depends on multiple factors including post-transplant marrow MRD status, cytogenetic risk, disease localization, and maintenance strategy (26).

An important distinction among MRD technologies is the maturity of the evidence supporting their clinical use. Bone marrow-based NGF and NGS currently have the strongest validation, as they are incorporated into consensus response criteria and supported by extensive clinical trial experience and meta-analytic evidence linking MRD negativity with improved PFS and OS. Functional imaging provides complementary information, particularly in patients with focal lesions or extramedullary disease, but should not be considered interchangeable with marrow-based MRD assessment. By contrast, mass spectrometry and ctDNA are promising minimally invasive tools for longitudinal surveillance, but their evidence base remains less mature. Available data derive largely from translational studies, prospective observational cohorts, retrospective analyses, or protocol-defined exploratory endpoints, rather than randomized trials demonstrating that treatment modification based on these assays improves clinical outcomes. Accordingly, mass spectrometry and ctDNA should currently be regarded as complementary or investigational approaches for risk refinement and disease surveillance, rather than as standalone tools for therapeutic decision-making outside prospective clinical trials.

Overall, current and emerging MRD platforms interrogate distinct biological compartments, and provide complementary information regarding tumor burden, spatial heterogeneity, and longitudinal disease evolution. Their different analytical characteristics, clinical applications, and limitations are summarized in **Table 1**. Together, these approaches support the transition from a single time-point assessment of residual disease toward a multidimensional monitoring framework integrating tumor biology, disease distribution, and host response.

**Table 1:** Current and emerging approaches for measurable residual disease assessment in multiple myeloma.

Method	Main compartment	Approximate sensitivity	Principal clinical value	Key limitations	Most appropriate use in dynamic monitoring	Evidence status/recommended use level
Next-generation flow cytometry (NGF)	Bone marrow aspirate	10^-5 to 10^-6	Rapid immunophenotypic identification of aberrant plasma cells; broadly applicable without the need for patient-specific baseline clonotype identification	Susceptible to hemodilution, patchy marrow infiltration, sample quality, and inter-laboratory variability if protocols are not standardized	Standard marrow-based MRD evaluation after induction, consolidation, transplantation, and during maintenance	*Validated clinical and trial-use platform*. Recommended for marrow-based MRD assessment when standardized protocols, adequate cellular input, and sample-quality controls are available
Next-generation sequencing (NGS)	Bone marrow aspirate	Up to 10^-6	Highly sensitive molecular tracking of clonal immunoglobulin rearrangements; strong reproducibility and extensive clinical-trial validation	Requires identification of a trackable baseline clonotype; turnaround time, cost, and platform availability may limit routine use	Deep molecular response assessment, serial monitoring, clinical trials, and MRD-adapted treatment strategies	*Validated clinical and trial-use platform*. Recommended for deep molecular MRD assessment, particularly in clinical trials and serial monitoring when a trackable baseline clonotype is available
Allele-specific oligonucleotide quantitative PCR (ASO-qPCR)	Bone marrow aspirate	10^-5 to 10^-6	Historically important molecular approach with high analytical sensitivity when patient-specific primers are feasible	Labor-intensive, technically demanding, affected by somatic hypermutation, and less standardized than NGF or NGS in multiple myeloma	Selected molecular monitoring settings, mainly where validated patient-specific assays are already available	*Historical/selected-use molecular method*. Useful in selected settings with validated patient-specific assays, but largely superseded by standardized NGF and NGS platforms
Functional imaging, including FDG-PET/CT and advanced PET approaches	Whole body	Not directly comparable with cellular assays	Detects focal bone lesions, metabolically active disease, and extramedullary involvement that may be missed by single-site marrow sampling	Limited sensitivity for microscopic disease; tracer uptake and interpretation may vary; false-negative lesions can occur	Complementary assessment of spatial disease heterogeneity, especially in patients with focal lesions, extramedullary disease, or marrow MRD negativity with clinical suspicion	*Clinically established complementary tool.* Recommended for spatial disease assessment, especially in focal, paramedullary, or extramedullary disease and in marrow/imaging discordance.
Mass spectrometry-based M-protein tracking	Peripheral blood or serum	Higher than conventional electrophoresis and immunofixation; assay-dependent	Minimally invasive serial monitoring of patient-specific monoclonal proteins; may detect residual disease beyond conventional serological response criteria	Not yet fully standardized for MRD-guided decision-making; persistence of circulating monoclonal protein may reflect protein half-life rather than viable residual tumor in some contexts	Longitudinal blood-based monitoring, particularly as a complement to marrow MRD and conventional serology	*Emerging complementary tool.* Promising for minimally invasive longitudinal monitoring, but not yet validated as a standalone MRD-guided decision tool
Circulating tumor DNA and circulating myeloma cell analysis	Peripheral blood or plasma	Assay-dependent; currently investigational for MRD-level detection	Minimally invasive systemic sampling; may capture clonal evolution, spatial heterogeneity, and extramedullary disease more broadly than iliac crest aspirates	Lower and variable sensitivity compared with marrow-based MRD in many studies; methodological heterogeneity; thresholds require prospective validation	Research setting, relapse prediction, clonal evolution monitoring, and integration into multimodal risk models	*Investigational/translational tool.* Promising for systemic disease tracking and clonal evolution, but requires prospective validation before routine MRD-guided treatment use
MRD assessment in autologous stem cell grafts	Peripheral blood stem cell product	Method-dependent	Provides additional information on tumor contamination before autologous stem cell transplantation and may refine pre-transplant risk assessment	Single-time-point assessment; graft contamination is prognostic but not necessarily the primary driver of relapse	Adjunctive risk factor in transplant-eligible patients, especially when interpreted alongside marrow MRD and baseline risk features	*Adjunctive risk marker*. May refine transplant-related risk assessment, but should be interpreted together with post-transplant marrow MRD and baseline disease biology

The table summarizes the main methodological platforms used or investigated for MRD evaluation, highlighting their biological compartment, approximate sensitivity, clinical contribution, key limitations, potential role in longitudinal disease monitoring, and evidence status or recommended use level. The final column distinguishes validated clinical and trial-use platforms, clinically established complementary tools, emerging complementary approaches, investigational methods, and adjunctive risk markers. ASO-qPCR, allele-specific oligonucleotide quantitative polymerase chain reaction; FDG-PET/CT, fluorodeoxyglucose positron emission tomography/computed tomography; MRD, measurable residual disease; NGF, next-generation flow cytometry; NGS, next-generation sequencing.

To translate these methodological considerations into a practical monitoring framework, **Box 1** presents provisional minimum standards for MRD assessment, imaging integration, sample handling, and immune profiling.

Box 1 **Provisional dynamic monitoring algorithm and minimum standards for MRD and immune profiling in multiple myeloma**

Purpose. This boxed algorithm provides a provisional framework for standardized MRD reporting, longitudinal monitoring, imaging integration, blood-based assessment, and layered immune profiling. It is intended to support clinical trial design, structured surveillance, and translational research. It should not be interpreted as a formal clinical guideline or a validated routine-care pathway.

**Step 1. Define the monitoring context**

◻ Newly diagnosed disease

◻ Post-induction assessment

◻ Post-ASCT assessment, when applicable

◻ Post-consolidation assessment

◻ Maintenance monitoring

◻ Suspected biochemical or clinical relapse

◻ Pre-de-escalation or treatment discontinuation within a clinical trial

MRD results should always be interpreted according to treatment phase, prior disease distribution, baseline risk, therapeutic modality, assay sensitivity, and sample quality.

**Step 2. Perform validated marrow-based MRD assessment**

Core requirement: bone marrow MRD assessment using next-generation flow cytometry (NGF) or next-generation sequencing (NGS).

◻ Report assay platform

◻ Report specimen type

◻ Report analytical sensitivity

◻ Report cellular input or sequencing input

◻ Report sample adequacy

◻ Use first-pull marrow aspirate whenever possible to reduce hemodilution

◻ Prefer serial assessment with the same validated platform

Minimum sensitivity: 10^-5.

Preferred sensitivity when feasible: 10^-6, particularly in clinical trials, deep-response assessment, and treatment-discontinuation studies.

**Suggested timing of serial MRD assessment:** Assess MRD after induction, after ASCT when applicable, and after consolidation. During maintenance or post-treatment surveillance, serial reassessment should occur at protocol-defined intervals and earlier when biochemical, clinical, imaging, or other biological findings raise concern for relapse. For the documentation of sustained MRD negativity, repeat assessments should be separated by at least 12 months.

Decision point A. Is marrow MRD negative?

If no: classify as persistent measurable disease and interpret it according to MRD kinetics, baseline risk, treatment phase, and immune status. Treatment intensification strategies should be considered only within clinical trials or predefined institutional protocols.

If yes: do not equate MRD negativity with cure. Assess durability, imaging status, sample quality, baseline disease biology, and immune competence.

**Step 3. Evaluate spatial disease with imaging when indicated**

PET/CT should be considered:

◻ At baseline when focal or extramedullary disease is suspected

◻ After therapy in patients with prior imaging-positive lesions

◻ In marrow MRD-negative patients with biochemical, clinical, or radiological concern

◻ Before treatment de-escalation or discontinuation in clinical trials.

Imaging should be interpreted as complementary to marrow MRD because it addresses spatial heterogeneity, focal residual disease, and extramedullary escape.

Decision point B. Is there discordance between marrow MRD and imaging?

Marrow MRD negative but PET/CT positive: classify as discordant residual disease and avoid treatment de-escalation outside a protocol-defined setting.

Marrow MRD positive but PET/CT negative: interpret as molecular or immunophenotypic residual disease and monitor MRD kinetics longitudinally.

**Step 4. Add minimally invasive monitoring when available**

Blood-based tools may include:

◻ Mass spectrometry-based monoclonal protein tracking

◻ Circulating tumor DNA

◻ Circulating myeloma-cell assessment

These approaches may support longitudinal surveillance and systemic disease assessment, but should regarded as complementary or investigational. They should not replace validated marrow-based MRD outside protocol-defined settings.

**Step 5. Assess immune competence**

Because evidence supporting immune profiling remains preliminary and platform-dependent, the following panel should be regarded as a translational framework for clinical trials and structured monitoring studies rather than algorithm for routine clinical care.

Core clinically feasible layer:

◻ Complete blood count with absolute lymphocyte count

◻ Quantitative immunoglobulins, including IgG, IgA, and IgM

◻ Flow-cytometric enumeration of CD3+, CD4+, CD8+, CD19+ B-cell, and CD16+/CD56+ NK-cell compartments

◻ CD4/CD8 ratio;

◻ Assessment of B-cell and humoral immune recovery

This layer provides broad measures of immune reconstitution, including lymphopenia, B-cell and NK-cell recovery, and hypogammaglobulinemia.

Expanded translational layer

◻ CD8+ T-cell differentiation subsets, including naïve, central memory, effector memory, and terminally differentiated compartments

◻ Selected exhaustion or activation markers, including PD-1, TIGIT, LAG-3, TIM-3, HLA-DR, and CD38

◻ Regulatory T cells, preferably phenotypically defined as CD4+CD25highCD127low with optional FOXP3 confirmation

◻ NK-cell activation and maturation markers, including NKG2D, NKG2A, CD16, CD57, and killer-cell immunoglobulin-like receptor patterns

◻ Selected myeloid suppressor-cell populations, including monocytic and granulocytic monocytic and granulocytic myeloid-derived suppressor cells when technically standardized

Research-oriented layer:

◻ CyTOF

◻ Single-cell RNA sequencing

◻ Spatial profiling or spatial transcriptomics

◻ T-cell receptor repertoire analysis

◻ Cytokine profiling

◻ Integrated multi-omic immune signatures

No individual immune marker should currently be used as a standalone therapeutic trigger. The purpose of this layered panel is to identify reproducible immune variables that may add prognostic or predictive value beyond MRD, imaging, and baseline risk.

Decision point C. Is MRD status concordant with immune competence?

***MRD negative, immune recovery, and negative imaging***: favorable dynamic profile; continue standard surveillance and consider treatment de-escalation only within prospective, protocol-defined clinical trials.

***MRD negative with immune impairment:*** discordant remission; consider closer monitoring, infection-risk assessment, and and longitudinal reassessment of MRD and immune recovery.

***MRD positive with favorable immune profile***: residual disease with possible partial immune control; interpret according to MRD kinetics, baseline risk, and treatment context. Consider immune-based consolidation only within clinical trials.

***MRD positive with immune dysfunction and/or imaging-positive disease***: high-risk persistence; consider clinical trial enrollment, intensification surveillance, and treatment adaptation according to the clinical setting and predefined protocols.

**Step 6. Define clinical actionability**

At present, MRD and immune profiling should support:

◻ Risk-adapted surveillance

◻ Biological interpretation of response and relapse risk

◻ Clinical trial stratification

◻ Rational design of intensification, de-escalation, or discontinuation studies

Treatment modification based on MRD or immune biomarkers should preferably occur within prospective clinical trials or predefined institutional protocols until validated therapeutic algorithms are available.

## 4 Clinical meaning of MRD: depth, timing, and dynamics

The clinical relevance of MRD in MM is supported by extensive evidence demonstrating its strong association with long-term outcomes. A large meta-analysis, including patients treated with contemporary treatment approaches, confirmed that MRD negativity is associated with significantly prolonged PFS and OS, independently of conventional response categories and across different treatment settings (8). These findings have fundamentally changed the interpretation of treatment response in MM, demonstrating that patients achieving CR are not biologically equivalent. While conventional response criteria define the absence of detectable monoclonal protein by standard laboratory methods, MRD assessment identifies a subgroup of patients with substantially deeper tumor reduction and a higher probability of prolonged disease control.

However, the prognostic meaning of MRD is not absolute and depends on several contextual variables, including assay sensitivity, timing of assessment, and durability of response. Analytical sensitivity is particularly relevant because a sample classified as negative at a threshold of 10^-5 may still contain detectable disease with methods reaching 10^-6 sensitivity or through complementary approaches such as functional imaging or blood-based biomarkers. Therefore, MRD negativity represents a measurement-dependent state rather than a universal biological definition of disease eradication. The timing of MRD assessment also influences its clinical interpretation. MRD negativity achieved after induction therapy, following ASCT MRD negativity, after consolidation, or during maintenance may represent different biological states and may carry distinct prognostic implications. Early clearance of residual disease reflects treatment sensitivity, whereas persistent negativity over time provides stronger evidence of durable disease control. In this context, sustained MRD negativity, generally defined as repeated negative assessments separated by at least 12 months, appears to provide superior prognostic discrimination compared with a single negative evaluation (27, 28).

Beyond a binary positive/negative classification, MRD should increasingly be considered a longitudinal biomarker reflecting the evolving interaction between treatment pressure, residual tumor biology, and host response. Persistent MRD positivity after intensive therapy identifies patients with a higher probability of relapse and may indicate incomplete disease control. Conversely, conversion from MRD positivity to negativity during consolidation or maintenance reflects ongoing tumor clearance and may identify patients achieving progressively deeper responses. Importantly, the reappearance of detectable disease after a period of MRD negativity may precede biochemical or clinical relapse, creating a potential window for early intervention strategies. Nevertheless, implementation of MRD-guided therapeutic decisions requires prospective validation, including harmonization of assay platforms, assessment schedules, and clinically meaningful thresholds for action (27).

The most challenging clinical scenario is relapse occurring despite previous documentation of MRD negativity. Importantly, this phenomenon does not undermine the value of MRD; rather, it highlights the biological and technical limitations of any single measurement strategy. Apparent MRD-negative relapse may result from insufficient sampling, hemodilution, inadequate cellular input, patchy marrow infiltration, or disease recurrence in unsampled anatomical compartments such as extramedullary sites. Beyond technical explanations, relapse after MRD negativity reflects the ability of residual malignant clones to persist below current detection thresholds, remain dormant, or exploit protective biological niches. Mechanisms including immune escape, antigen modulation, altered stromal interactions, metabolic adaptation, and therapy-driven clonal selection may contribute to the re-emergence of clinically relevant disease despite previous deep responses. Therefore, MRD negativity should be interpreted as a powerful prognostic modifier rather than as a surrogate for cure. Its true clinical meaning depends on the depth, timing, durability, spatial distribution of disease, and biological context in which residual tumor cells persist or are eliminated (10, 28).

## 5 MRD as a biological trait rather than a purely quantitative state

An emerging conceptual framework proposes that MRD should not be interpreted solely as a quantitative estimate of remaining tumor burden, but also as a biological characteristic reflecting the ability of residual malignant clones to persist, adapt, and eventually regenerate clinically significant disease. This perspective is particularly relevant in MM, where residual plasma cells exist within a complex ecosystem shaped by tumor evolution, bone marrow microenvironmental interactions, immune surveillance, and therapeutic pressure. Accordingly, MRD positivity or negativity alone may not fully capture the biological properties of residual disease. Two patients with comparable levels of detectable residual tumor may experience different trajectories depending on whether persisting clones remain biologically constrained by effective immune control or acquire features associated with treatment resistance and relapse (10).

This biological interpretation of MRD can be understood through several interconnected mechanisms that determine the fate of residual malignant populations. Clonal fitness reflects the capacity of residual plasma-cell clone to survive under therapeutic pressure and acquire proliferative advantage during disease evolution. Phenotypic plasticity allows malignant cells to modify antigen expression, differentiation state, and transcriptional programs, potentially influencing both their detectability and their sensitivity to immune- and drug-mediated elimination. Metabolic adaptability enables persistence within hypoxic, nutrient-limited and otherwise hostile marrow environments by allowing tumor cells to reprogram energy utilization and stress responses. Immune evasion encompasses multiple mechanisms, including impaired antigen presentation, altered checkpoint pathways, escape from NK-cell surveillance, and suppression of cytotoxic T-cell activity. Finally, microenvironmental dependence highlights the role of stromal interactions, cytokine signaling, osteoclast activity, and protective marrow niche in supporting residual tumor survival and mediating drug resistance (29–31).

This biological-trait model provides a framework for interpreting several clinical observations that cannot be fully explained by a purely quantitative approach. It may explain why some patients with persistent MRD positivity experience prolonged disease stability, why some individuals relapse despite achieving MRD negativity, why marrow-based and imaging-based assessments provide discordant results, and why the characteristics of residual disease after immune-based therapies may differ from those observed after conventional cytotoxic or proteasome inhibitor-contain regimens. In this context, the clinically relevant question is evolving from “is MRD detectable?” toward more biologically informative questions: what type of residual disease persists, where is it located, how does it interact with the immune microenvironment, and is the host capable of maintaining long-term control (9)?

## 6 Immune profiling in multiple myeloma: from microenvironmental biology to disease monitoring

MM develops within a highly dynamic bone marrow microenvironment in which malignant plasma cells coexist with and actively remodel multiple immune and stromal compartments. Rather than representing a passive background, the immune microenvironment functions as a critical regulator of tumor persistence, therapeutic response, and disease evolution (32). Malignant plasma cells interact with a complex network of stromal cells, osteoclasts, macrophages, dendritic cells, regulatory T cells, myeloid-derived suppressor cells, NK cells, and cytotoxic T cells. These interactions promote tumor-cell survival, induce immune dysfunction, provide protection from therapeutic pressure, and facilitate the establishment of resistant disease niches (33). Importantly, immune alterations are not restricted to advanced disease but are already detectable during precursor stages progressively evolving from monoclonal gammopathy of undetermined significance and smoldering MM toward symptomatic disease (34, 35).

High-dimensional immune studies have demonstrated that immune composition carries prognostic and response-associated information that is not captured by conventional disease classifications. In a deep phenotyping study of 94 patients, 32 immune-cell subsets were analyzed in matched bone marrow and peripheral blood samples. The study showed that peripheral blood does not accurately reproduce the immune landscape of the bone marrow compartment, that adverse ISS stage and high-risk cytogenetics are associated with distinct immune signatures and that MRD-negative patients exhibit a more mature and functionally experienced CD4+ and CD8+ T-cell phenotype (36). These observations provide a biological framework linking MRD status with immune context. MRD negativity is not simply the consequence of effective tumor-directed therapy but may also reflect the presence of a more competent immune environment capable of maintaining long-term disease control. Conversely, patients with comparable levels of residual disease may have different outcomes depending on whether immune surveillance is preserved or profoundly impaired.

However, the evidence supporting individual immune markers as prognostic or predictive tools remains heterogeneous. Most immune profiling data in MM derive from translational cohorts, correlative analyses, immune reconstitution studies, single-cell studies, or exploratory analyses embedded within clinical trials, rather than from randomized biomarker-driven studies. Accordingly, candidate markers—including CD8+ T-cell differentiation, T-cell exhaustion signatures, NK-cell recovery, regulatory T-cell frequency, myeloid suppressor populations, and humoral immune reconstitution—should currently be interpreted as biologically informative risk modifiers rather than validated standalone biomarkers for therapeutic decision-making. Their clinical utility will require prospective validation, harmonized assays, standardized thresholds, and evidence that immune-informed interventions improve patient outcomes.

Single-cell technologies have further expanded understanding of immune evolution in MM by resolving cellular states that are not apparent through conventional immunophenotyping. Single-cell RNA sequencing studies in precursor conditions and overt MM have identified early alterations in cytotoxic lymphocyte compartments, including depletion of granzyme K-positive memory cytotoxic T cell populations, abnormal NK-cell states, and dysregulated antigen-presentation pathways in CD14-positive monocytes. These findings suggest that immune dysfunction develops early during disease evolution and may contribute to progression, therapeutic resistance, and relapse (37).

Immune reconstitution after therapy is another emerging dimension of disease monitoring. Following ASCT, recovery of lymphocyte subsets, restoration of T-cell repertoire diversity, NK-cell expansion and functional recovery, and normalization of inflammatory pathways may influence durability of response (38, 39). Conversely, immune exhaustion after transplantation, characterized by impaired T-cell function and altered immune repertoire, may create a permissive environment for relapse and influence the optimal timing of subsequent immunotherapeutic interventions (40).

The immune consequences of modern therapies further reinforce the need for dynamic immune monitoring. Anti-CD38 monoclonal antibodies exert direct anti-myeloma activity but also reshape immune architecture by eliminating CD38-positive immunoregulatory populations, promoting T-cell expansion, and altering T-cell receptor repertoire composition (41). However, prolonged immune perturbation may also contribute to infectious complications and altered immune recovery.

In the era of T-cell redirection, including CAR T-cell therapy and bispecific antibodies, immune profiling has become increasingly relevant because therapeutic efficacy depends not only on tumor characteristics but also on the quality of the host immune substrate. Baseline T-cell fitness, exhaustion status, antigen density, previous treatment exposure, and inflammatory context may influence T-cell expansion, persistence, toxicity, and patterns of relapse. Therefore, immune profiling has the potential to evolve from a descriptive prognostic tool into a predictive biomarker capable of guiding patient selection and therapeutic sequencing in immunotherapy-based strategies (42).

## 7 Integrating MRD and immune profiling in the immunotherapy era

The success of immunotherapy amplifies the need to integrate MRD and immune profiling. Anti-CD38 quadruplets have improved response depth in newly diagnosed MM. In GRIFFIN, addition of daratumumab to RVd increased the depth of response and MRD negativity in transplant-eligible patients (43). In PERSEUS, daratumumab plus VRd followed by daratumumab-lenalidomide maintenance significantly improved PFS and produced higher MRD-negative rates than VRd alone, with MRD assessed by NGS according to IMWG criteria (44). These studies illustrate a fundamental change in MM management: when a large proportion of patients achieve deep molecular responses, the clinical question is no longer simply whether MRD negativity has been achieved, but whether such remission is biologically stable. The ability to distinguish durable immune-controlled remission from vulnerable MRD-negative states will become increasingly important as treatment strategies move toward response-adapted approaches.

The same concept applies even more strongly to T-cell redirecting therapies. CAR T-cell therapies have demonstrated unprecedented activity in relapsed/refractory MM, with substantial proportions of heavily pretreated patients achieving deep responses. Idecabtagene vicleucel produced durable responses in the KarMMa study, including high rates of MRD negativity among patients achieving complete response or better (45). Ciltacabtagene autoleucel produced deep and durable responses in CARTITUDE-1 (46). Teclistamab, elranatamab, and talquetamab have demonstrated clinically meaningful activity in triple-class-exposed disease by redirecting T cells against BCMA or GPRC5D (47–49). These agents can induce rapid MRD clearance, but they also expose the dependence of response durability on T-cell fitness, antigen persistence, immune exhaustion, and infection-related morbidity.

However, these therapies also reveal the limitations of interpreting response through MRD alone. Following immune-mediated tumor elimination, MRD negativity may have a different biological meaning compared to MRD negativity after conventional chemotherapy, ASCT, or maintenance therapy. Immune-mediated responses depend on the persistence and functionality of effector cells, antigen availability, and the absence of mechanisms of immune escape. Thus, an MRD-negative patient after T-cell redirection may still present profound immune perturbations, including T-cell exhaustion, impaired immune reconstitution, hypogammaglobulinemia, B-cell depletion, or antigen-negative relapse risk. Conversely, detectable low-level MRD after immunotherapy may not uniformly predict imminent progression if residual clones remain under effective immune control (50, 51).

These considerations support a composite model in which MRD status is interpreted together with immune competence, imaging findings, and disease kinetics. Such an approach may identify biologically distinct patient categories that are not captured by conventional response criteria. Patients with sustained MRD negativity, immune recovery, and absence of imaging abnormalities may represent candidates for treatment de-escalation or discontinuation strategies within clinical trials. Conversely, MRD negativity accompanied by persistent immune dysfunction may require continued maintenance, enhanced infection surveillance, or closer biological monitoring. Patients with persistent MRD but favorable immune characteristics may represent a population in whom immune-based approaches can still achieve progressive disease control, whereas MRD persistence associated with immune dysfunction, adverse genomics, or imaging-positive disease may justify therapeutic intensification.

A dynamic monitoring framework integrating MRD kinetics, immune competence, imaging status, and circulating biomarkers may therefore provide a more biologically accurate definition of relapse risk than any individual parameter alone (15).

**Figure 2:**
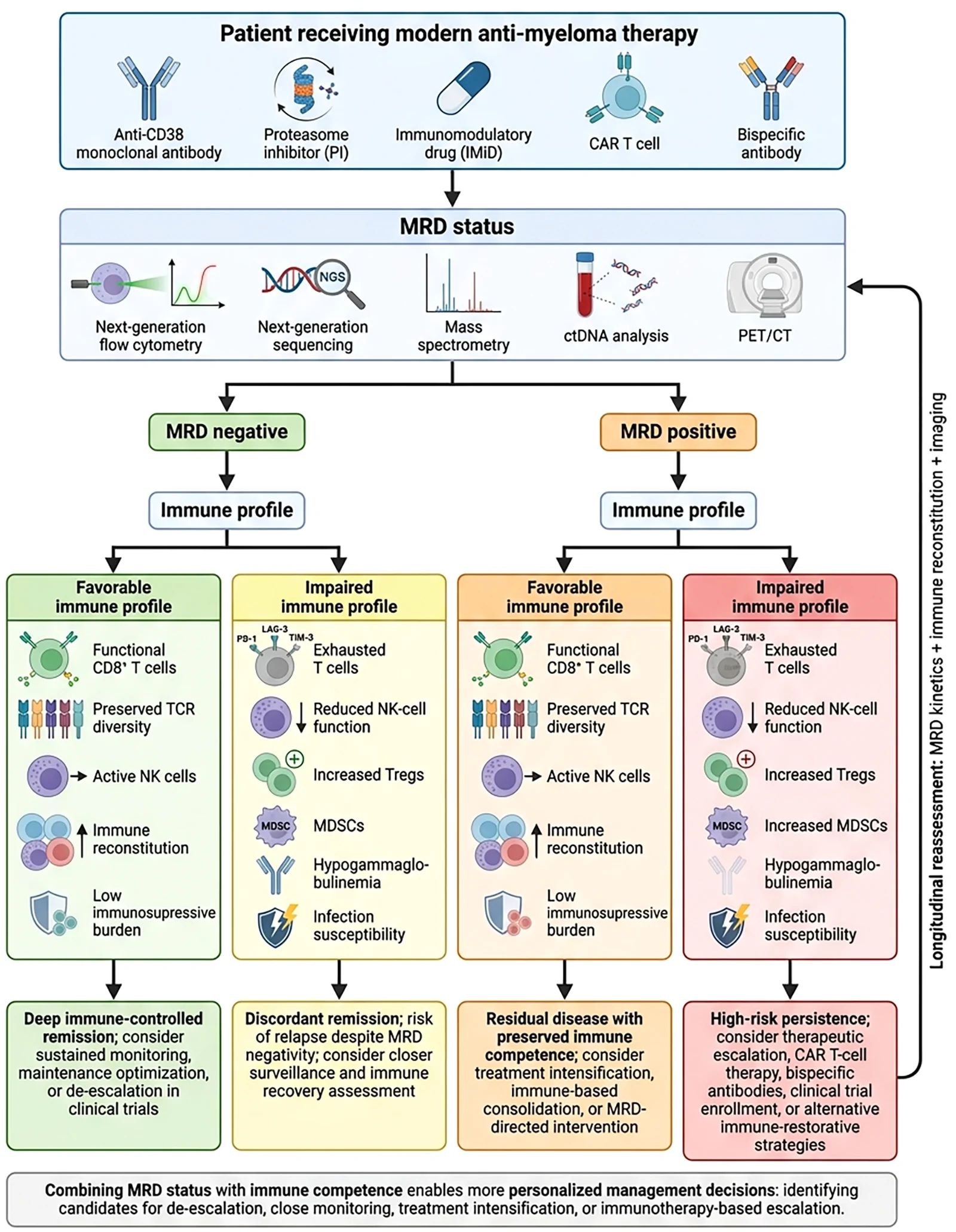
Integrated MRD and immune profiling-guided therapeutic framework in multiple myeloma. Measurable residual disease assessment provides a sensitive estimate of residual tumor burden, whereas immune profiling captures the functional status of host anti-myeloma surveillance. Integrating these two dimensions may identify biologically distinct clinical states. Patients with MRD negativity and favorable immune reconstitution may represent candidates for treatment optimization or de-escalation within clinical trials, whereas MRD-negative patients with immune dysfunction may require closer surveillance because relapse can occur despite undetectable disease. Persistent MRD positivity, particularly when associated with T-cell exhaustion, impaired NK-cell activity, expanded immunosuppressive populations, or infection-prone immune suppression, may define a high-risk state requiring therapeutic intensification, immune-based consolidation, or enrollment in clinical trials. Longitudinal reassessment is essential because both MRD status and immune competence evolve under treatment pressure. MRD, Measurable Residual Disease; CD38, Cluster of Differentiation 38; PI, Proteasome Inhibitor; IMiD, Immunomodulatory Drug; CAR T cell, Chimeric Antigen Receptor T cell; NGS, Next-Generation Sequencing; ctDNA, Circulating Tumor DNA; PET/CT, Positron Emission Tomography/Computed Tomography; TCR, T-Cell Receptor; NK cells, Natural Killer cells; PD-1, Programmed Cell Death Protein 1; LAG-3, Lymphocyte-Activation Gene 3; TIM-3, T-cell Immunoglobulin and Mucin-domain containing-3; Tregs, Regulatory T cells; MDSCs, Myeloid-Derived Suppressor Cells.

The proposed integration of MRD and immune profiling to guide response-adapted therapeutic strategies is summarized in **Table 2** and illustrated in **Figure 2**.

**Table 2:** Integrated interpretation of MRD status and immune profiling in multiple myeloma.

Dynamic disease state	Biological interpretation	Immune features to assess	Prognostic implication	Potential clinical implication
Sustained MRD negativity with favorable immune reconstitution	Deep tumor reduction accompanied by preserved or restored host immune surveillance	Recovery of functional CD8+ T cells, preserved T-cell receptor diversity, active NK-cell compartment, low Treg and MDSC burden, absence of dominant exhaustion phenotype	Most favorable dynamic profile; associated with durable disease control and prolonged remission	Continue standard monitoring; consider maintenance optimization or de-escalation only within validated clinical-trial frameworks
Sustained MRD negativity with impaired immune profile	Residual tumor burden is below assay detection limits, but immune surveillance remains biologically fragile	T-cell exhaustion, reduced NK-cell cytotoxic potential, persistent immunosuppressive myeloid populations, expanded Tregs, hypogammaglobulinemia, infection susceptibility	Discordant remission state; relapse may still occur despite undetectable marrow MRD	Closer surveillance; repeat MRD assessment; evaluate immune recovery, infection risk, and need for maintenance adaptation
MRD conversion from negative to positive	Re-emergence of measurable clonal disease after a previous deep response	Rising residual tumor signal with possible parallel immune exhaustion or loss of immune control	Early warning state; often precedes biochemical or clinical relapse	Confirm with repeat marrow MRD and/or imaging; consider pre-emptive treatment intensification in clinical trials
Persistent MRD positivity with favorable immune competence	Detectable residual disease persists, but anti-myeloma immune function may still contribute to partial disease control	Functional cytotoxic T cells, preserved NK-cell activity, limited immunosuppressive expansion, absence of severe T-cell exhaustion	Intermediate-risk state; outcome depends on MRD kinetics, baseline risk, and treatment context	Consider consolidation, immune-based intensification, or MRD-directed intervention according to clinical setting
Persistent MRD positivity with impaired immune profile	Residual tumor burden persists in a permissive immune microenvironment	Exhausted PD-1/LAG-3/TIM-3-positive T cells, dysfunctional NK cells, increased Tregs, increased MDSCs, inflammatory or suppressive cytokine profile	High-risk dynamic profile; increased likelihood of progression, immune escape, and treatment resistance	Therapeutic escalation, clinical-trial enrollment, CAR T-cell therapy, bispecific antibodies, or alternative immune-restorative approaches may be appropriate
Marrow MRD negativity with PET-positive or liquid biopsy-positive disease	Discordance suggests spatial heterogeneity, extramedullary escape, or residual disease outside the sampled marrow compartment	Immune profiling should be interpreted with imaging and circulating biomarkers rather than marrow MRD alone	Higher-risk state than marrow MRD negativity alone; may explain relapse despite apparent deep response	Integrate PET/CT, ctDNA or circulating myeloma cell analysis, and repeat longitudinal assessment before treatment de-escalation

This table proposes a clinically oriented framework for interpreting MRD results in relation to immune competence, spatial disease assessment, and longitudinal disease kinetics. The aim is not to replace validated MRD criteria, but to contextualize MRD status within a broader tumor–host ecosystem that may better identify patients suitable for standard monitoring, intensified surveillance, therapeutic escalation, or trial-based de-escalation. ctDNA, circulating tumor DNA; MDSC, myeloid-derived suppressor cell; MRD, measurable residual disease; NK, natural killer; PET/CT, positron emission tomography/computed tomography; Treg, regulatory T cell.

## 8 MRD-guided therapy: evidence, uncertainty, and current trial direction

MRD-guided therapy is one of the most clinically relevant applications of dynamic disease monitoring in MM. The underlying rationale is intuitive: patients with persistent measurable disease may benefit from therapeutic intensification, whereas patients achieving deep and sustained remission may avoid unnecessary exposure to treatment-related toxicity, financial burden, and cumulative immune impairment. However, despite the strong prognostic value of MRD, its use as a therapeutic decision-making tool remains an evolving field. The key distinction is that MRD is an established biomarker of outcome, whereas the ability of MRD-directed interventions to improve survival compared with standard strategies is still being prospectively defined (27).

The evolving regulatory landscape further illustrates the translational relevance of MRD in MM. In January 2026, the U.S. Food and Drug Administration (FDA) issued draft guidance on the use of MRD and complete response as primary endpoints to support accelerated approval of drugs and biological products for MM. For the purposes of this guidance, the MRD endpoint is defined as the bone marrow MRD negativity rate, assessed by flow cytometry- or sequencing-based methods, among patients who have achieved complete response or stringent complete response (52). Importantly, the document is draft guidance, is not for implementation in its current form, and contains non-binding recommendations. In Europe, the European Medicines Agency (EMA) reflection paper considers the use of MRD as an intermediate efficacy endpoint in controlled randomized clinical studies designed to demonstrate benefit on clinically meaningful hard endpoints (53). Together, these documents reflect increasing regulatory engagement with MRD as a trial endpoint, while emphasizing that its regulatory use remains context-dependent and requires assay validation, appropriate sensitivity and timing of assessment, robust trial design, and confirmatory evidence of long-term clinical benefit.

The MASTER trial provided one of the first prospective demonstrations that MRD response could be incorporated into a treatment-adaptation strategy. Patients with newly diagnosed MM received daratumumab, carfilzomib, lenalidomide, and dexamethasone, followed by ASCT, and consolidation. Patients achieving sustained MRD negativity were allowed to discontinue therapy and undergo observation with structured MRD surveillance (54). This study established the feasibility of a finite, response-adapted treatment paradigm and demonstrated that MRD surveillance could support selective treatment discontinuation. However, outcomes were less favorable among patients with multiple high-risk cytogenetic abnormalities, emphasizing a fundamental principle of dynamic risk assessment: MRD status cannot be interpreted independently from baseline genomic risk and disease biology.

More recent MRD-directed studies illustrate both the promise and the current limitations of treatment adaptation based on MRD. In a prospective study of 52 patients who discontinued lenalidomide maintenance after achieving sustained bone marrow and imaging MRD negativity for three years after ASCT (55). After a median follow-up of three years from maintenance discontinuation, the 3-year treatment-free survival rate was 75.8%, whereas the 3-year progression-free survival rate was 92.9%. Twelve patients (23%) converted from MRD negativity to MRD positivity and restarted lenalidomide maintenance, while four patients (7.6%) experienced disease progression. However, the study lacked a randomized comparator arm and enrolled a highly selected population with durable marrow and imaging MRD-negative remission; therefore, its findings cannot establish the safety or efficacy of maintenance discontinuation for the broader myeloma population, particularly for patients with high-risk disease. Accordingly, the study supports the feasibility of MRD-informed maintenance discontinuation in carefully selected patients with close longitudinal monitoring, but does not establish discontinuation as a broadly applicable standard strategy.

The phase 3 MIDAS trial provides an important randomized example of MRD-guided consolidation after six cycles of isatuximab, carfilzomib, lenalidomide, and dexamethasone (Isa-KRd) induction in transplant-eligible patients with newly diagnosed M (56). M. Among patients who were MRD-negative at 10^-5 after induction, MRD negativity at 10^-6 before maintenance was achieved in 86% of patients assigned to ASCT followed by two cycles of Isa-KRd and in 84% of those assigned to six additional cycles of Isa-KRd, with no significant difference between groups. Among patients who remained MRD-positive at 10^-5 after induction, tandem ASCT did not improve the pre-maintenance MRD-negativity rate at 10^-6 compared with single ASCT followed by two cycles of Isa-KRd (32% vs 40%, respectively). These results demonstrate that MRD status can be used to stratify patients and test response-adapted consolidation strategies; however, they also caution against assuming that MRD-directed escalation or de-escalation will necessarily improve long-term clinically meaningful outcomes.

Taken together, the MASTER trial, prospective maintenance-discontinuation studies, the randomized MIDAS trial, and ongoing MRD-directed studies demonstrate that MRD can be operationalized to stratify patients and evaluate response-adapted treatment strategies. However, the feasibility, safety, and clinical benefit of MRD-guided intervention remain context-specific and require prospective validation for each specific decision, including treatment intensification, transplant strategy, maintenance duration, and treatment discontinuation. MRD should therefore be considered an established prognostic biomarker and trial-stratification tool, whereas MRD-directed treatment modification remains an evolving investigational strategy rather than a routine standard of care.

Contemporary trials are now testing whether MRD can guide major therapeutic decisions such as transplantation, consolidation intensity, maintenance duration, and incorporation of immune-based therapies. The 2026 Trial Watch review highlighted several emerging paradigms, including immune consolidation with CAR T cells or bispecific antibodies, MRD-guided therapy through studies such as MASTER-2 and MIDAS, finite-duration treatment strategies, multi-antigen targeting, and early immune intervention approaches. MIDAS, for example, evaluates MRD-guided consolidation after induction in transplant-eligible disease, while MASTER-2 investigates whether bispecific antibodies can deepen responses beyond conventional MRD-adapted approaches (57). These trials reflect a broader evolution in therapeutic philosophy. However, the central unresolved question is whether MRD should function as an independent therapeutic trigger or whether it should be integrated into a multidimensional decision model incorporating immune recovery, imaging status, cytogenetic risk, and molecular evolution.

At present, MRD-guided treatment modification should be regarded as strongly evidence-supported for clinical-trial design and prognostic counseling, but not yet universally standardized for routine practice. Key uncertainties include the optimal threshold, the required duration of negativity, the role of 10^-6 versus 10^-5 sensitivity, the management of discordant marrow and imaging results, and the actionability of MRD conversion in the absence of biochemical relapse (15). These uncertainties are not reasons to delay MRD assessment; rather, they argue for structured monitoring protocols and prospective trials that test predefined actions.

## 9 Computational models and multidimensional risk prediction

The increasing complexity of MM monitoring will require analytical approaches capable of integrating longitudinal and heterogeneous biological information. As disease assessment moves beyond single time-point measurements toward serial evaluation of tumor burden, immune competence, imaging findings, and molecular evolution, computational models may provide the framework necessary to transform multiple biomarkers into clinically meaningful risk estimates.

A 2026 dynamic biomarker-based machine learning study represents an example of this emerging approach. The investigators analyzed 662 newly diagnosed MM patients, by integrating peripheral blood lymphocyte subsets, cytokine profiles, and bone marrow plasma-cell phenotypes assessed at diagnosis and longitudinally during treatment. Conventional prognostic models, including cytogenetic risk classification and ISS/R-ISS staging, showed limited ability to predict short-term treatment response beyond early treatment phases, whereas dynamic biomarkers such as CD8-positive T cells, CD56-positive NK cells, lymphocyte counts, and plasma-cell surface markers provided additional predictive information. At cycle 4, the biomarker-based model achieved an F1 score of 0.75 compared with 0.32 for R-ISS, illustrating the potential advantage of continuously updated biological assessment over static baseline classification (58).

This study is important because it operationalizes the central thesis of dynamic monitoring: serial, biologically informative measures can outperform baseline categories when the clinical task is to predict near-term response. However, such computational models require external validation, harmonized assays, interpretability, and prospective evaluation before clinical implementation. Machine learning should not be considered as a black-box replacement for clinical judgment, but rather a tool capable of integrating multiple partially informative signals into interpretable risk assessments (59, 60).

## 10 Proposed investigational integrated monitoring framework

The framework proposed here should be interpreted as a translational and investigational model rather than as an established clinical algorithm. Its purpose is to organize biologically relevant information— including MRD kinetics, imaging findings, immune competence, circulating biomarkers, and baseline risk—into a structured approach for biological interpretation, clinical trial design, and risk-adapted surveillance. It is not intended to direct routine treatment intensification, de-escalation, or discontinuation outside prospective trials or predefined institutional protocols.

A practical dynamic monitoring framework should be layered rather than dependent on a single assay. At diagnosis, baseline risk stratification should include ISS/R-ISS or R2-ISS, cytogenetics including 1q abnormalities, extramedullary disease assessment, renal and bone disease burden, frailty, and treatment eligibility. During treatment, response assessment should incorporate conventional serology, marrow MRD by a validated platform, imaging when clinically indicated or when prior focal disease is present, and immune markers that can be measured reproducibly. During maintenance or after finite therapy, monitoring should shift from response depth alone to response durability, MRD conversion, immune reconstitution, infection risk, and clinical relapse biology (61).

Given the evidence supporting immune profiling remains preliminary and platform-dependent, the proposed panel should be regarded as a translational framework for clinical trials and structured monitoring studies, rather than a validated algorithm. Immune profiling for routine practice is therefore not yet standardized, and no immune panel has been validated to replace established prognostic models, MRD assessment, or imaging. Nevertheless, a pragmatic immune monitoring panel can be organized into three layers (36, 42, 62).

The core clinically feasible layer should include complete blood count with absolute lymphocyte count, quantitative immunoglobulins, CD3+, CD4+, CD8+, CD19+ B-cell, and CD16+/CD56+ NK-cell enumeration, CD4/CD8 ratio, and assessment of humoral immune recovery. This layer captures broad immune reconstitution, lymphopenia, B-cell recovery, NK-cell recovery, and hypogammaglobulinemia, which are clinically relevant in patients receiving anti-CD38 antibodies, cellular therapies, or bispecific antibodies.

An expanded translational layer, suitable for referral centers and clinical trials, may include CD8+ T-cell differentiation subsets, effector-memory and terminally differentiated T-cell compartments, selected exhaustion or activation markers such as PD-1, TIGIT, LAG-3, TIM-3, HLA-DR, and CD38, regulatory T cells defined as CD4+CD25highCD127low with or without FOXP3, NK-cell activation and maturation markers such as NKG2D, CD16, CD57, NKG2A, and killer-cell immunoglobulin-like receptors, and selected myeloid suppressor-cell populations (36, 42).

A research-oriented layer may include CyTOF, single-cell RNA sequencing, spatial transcriptomics, T-cell receptor repertoire analysis, cytokine profiling, and integrated multi-omic immune signatures. These approaches may clarify mechanisms of immune escape, T-cell dysfunction, antigen-driven selection, and microenvironmental protection, but they require analytical harmonization and prospective validation before clinical implementation (62). The purpose of this layered panel is not to create an excessively complex immune score, but to identify reproducible immune variables that add prognostic or predictive value beyond MRD, imaging, and baseline risk (42, 62). Immune profiling is not yet standardized for routine clinical practice, and no immune panel has been validated to replace established prognostic models, MRD assessment, or imaging. Nevertheless, a pragmatic immune-monitoring panel can be organized into three layers (36, 42, 62).

The core, clinically feasible layer should include a complete blood count with absolute lymphocyte count; quantitative immunoglobulins; flow-cytometric enumeration of CD3+, CD4+, CD8+, CD19+ B-cell, and CD16+/CD56+ natural killer (NK)-cell compartments; the CD4/CD8 ratio; and assessment of B-cell and humoral immune recovery. This layer captures broad measures of immune reconstitution, including lymphopenia, B-cell and NK-cell recovery, and hypogammaglobulinemia, which are clinically relevant in patients receiving anti-CD38 antibodies, cellular therapies, or bispecific antibodies.

An expanded translational layer, suitable for referral centers and clinical trials, may include CD8+ T-cell differentiation subsets; effector-memory and terminally differentiated T-cell compartments; selected exhaustion or activation markers, including PD-1, TIGIT, LAG-3, TIM-3, HLA-DR, and CD38; regulatory T cells, phenotypically defined as CD4+CD25^high^CD127^low^ cells, with optional FOXP3 confirmation; NK-cell activation and maturation markers, including NKG2D, CD16, CD57, NKG2A, and killer-cell immunoglobulin-like receptor patterns; and selected myeloid suppressor-cell populations (36, 42).

A research-oriented layer may include CyTOF, single-cell RNA sequencing, spatial transcriptomics, T-cell receptor repertoire analysis, cytokine profiling, and integrated multi-omic immune signatures. These approaches may clarify mechanisms of immune escape, T-cell dysfunction, antigen-driven selection, and microenvironmental protection, but require analytical harmonization and prospective validation before clinical implementation (62). The purpose of this layered panel is not to create an excessively complex immune score, but to identify reproducible immune variables that add prognostic or predictive value beyond MRD, imaging, and baseline risk (42, 62).

The implementation of multidimensional monitoring must also consider cost, reimbursement, laboratory infrastructure, and feasibility outside academic or highly specialized centers. Broad access to NGF or NGS, serial PET/CT, mass spectrometry, ctDNA platforms, and multiparameter immune profiling is not uniform across healthcare systems. A scalable approach may therefore be preferable to a single universal model. In routine or resource-limited settings, a core monitoring layer could include standardized serological response assessment, marrow MRD by a validated NGF or NGS platform when available, imaging according to baseline disease distribution and clinical indication, and basic immune recovery markers such as absolute lymphocyte count, lymphocyte subsets, and quantitative immunoglobulins. An expanded layer, more feasible in referral centers or clinical trials, could include serial marrow MRD at predefined time points, PET/CT integration for patients with focal or extramedullary disease, deeper immune phenotyping, and selected blood-based tools. Finally, an investigational layer could incorporate mass spectrometry, ctDNA, single-cell technologies, spatial profiling, and computational risk modeling. Such a tiered strategy preserves the biological rationale of integrated monitoring while acknowledging real-world variability in access, cost, reimbursement, and technical expertise. The implementation of multidimensional monitoring must also consider cost, reimbursement, laboratory infrastructure, and feasibility outside academic or highly specialized centers. Access to NGF or NGS, serial PET/CT, mass spectrometry, ctDNA platforms, and multiparameter immune profiling remains uneven across healthcare systems. A scalable, tiered approach may therefore be more appropriate than a single universal model.

In routine or resource-limited settings, a core monitoring layer could include standardized serological response assessment; marrow MRD assessment using a validated NGF or NGS platform, when available; imaging guided by baseline disease distribution and clinical indication; and basic markers of immune recovery, including absolute lymphocyte count, lymphocyte subsets, and quantitative immunoglobulins. An expanded layer, more feasible in referral centers or clinical trials, could include serial marrow-based MRD assessment at predefined time points, PET/CT integration for patients with focal or extramedullary disease, expanded immune phenotyping, and selected blood-based monitoring tools. Finally, an investigational layer could incorporate mass spectrometry, ctDNA, single-cell technologies, spatial profiling, and computational risk modeling. This tiered strategy preserves the biological rationale for integrated monitoring while acknowledging real-world variation in access, cost, reimbursement, laboratory capacity, and technical expertise.

Together, **Table 1**; **Box 1**; **Table 2**; **Figures 1**, **2** provide a structured framework for translating dynamic disease monitoring into trial-oriented and clinically interpretable scenarios. This model is intentionally conservative and scalable: treatment modification should occur within clinical trials or carefully defined institutional protocols until prospective evidence confirms that immune-informed MRD decisions improve outcomes.

## 11 Future perspectives

The future of MM monitoring will likely depend on the transition from isolated biomarker assessment toward integrated, longitudinal, and biologically informed surveillance. As therapeutic strategies continue to induce increasingly deep responses, the challenge will no longer be only to determine whether disease is detectable, but to understand whether remission is durable, biologically stable, and supported by effective immune control.

A priority will be the harmonization of MRD assessment across platforms and clinical contexts. Although MRD negativity has become one of the strongest prognostic biomarkers in MM, interpretation requires consideration of assay sensitivity, sample quality, timing of evaluation, previous disease distribution, and the compartment being analyzed. A binary positive/negative classification may therefore be insufficient unless accompanied by information regarding analytical sensitivity, kinetics, and biological context (7, 10, 27).

Second, minimally invasive approaches are expected to become increasingly relevant for longitudinal monitoring. Serum mass spectrometry and ctDNA may enable more frequent assessment, reduce reliance on repeated invasive marrow sampling, and provide complementary information regarding systemic disease heterogeneity. Rather than replacing marrow-based MRD, these approaches will likely function as complementary tools that improve continuous surveillance and facilitate earlier detection of biological relapse (24, 25).

Third, immune biomarkers must be standardized and clinically validated. The most relevant immune parameter is unlikely to be a single marker such as PD-1 or CD8-positive T-cell count, but rather a composite immune signature integrating effector function, exhaustion pathways, regulatory suppression, humoral recovery, and inflammatory status (28, 36).

Fourth, MRD-guided de-escalation will require carefully defined safety boundaries. Sustained MRD negativity may support finite-duration strategies in selected patients, but treatment discontinuation cannot be considered independently from baseline genomic, extramedullary disease, immune competence, and the durability of remission (27, 63).

Finally, the concept of cure or functional cure in MM will likely require redefinition in the immunotherapy era. Durable treatment-free remission may become achievable for a subset of patients, but this objective will require more than deep tumor reduction. It will likely depend on sustained MRD negativity, absence of spatially undetected disease, restoration of immune surveillance, and continued protection against late immune escape (64, 65).

Future clinical trials should therefore move beyond response rates and traditional survival endpoints alone, incorporating integrated biological outcomes that include MRD kinetics, imaging-defined disease control, immune recovery, infection risk, quality of life, and treatment-free survival. Ultimately, the future of MM monitoring will likely depend on the ability to continuously characterize the interaction between residual tumor cells and host immunity, transforming risk assessment from a static classification system into a dynamic model of disease evolution.

## 12 Integrated perspectives: toward dynamic, biology-driven monitoring in multiple myeloma

The prognostic paradigm of MM is progressively shifting from a static model based predominantly on baseline disease characteristics toward a dynamic and multidimensional framework that incorporates treatment response, residual disease biology, and host immune competence. Baseline staging systems, including ISS, R-ISS, and R2-ISS, remains essential for initial risk assessment, clinical decision-making, and trial stratification; however, they provide only a snapshot of disease biology at diagnosis and cannot fully capture clonal evolution, treatment-induced selection pressure, immune remodeling, or the durability of response over time (7). MRD has become the central tool for measuring depth of response, yet MRD results are biologically meaningful only when interpreted in context (27). The context includes assay sensitivity, sample quality, imaging findings, baseline and functional risk, treatment modality, and the patient’s immune state (28).

Immune profiling is not ready to replace established prognostic markers, but it can address questions that MRD alone cannot answer (36). It can help explain why some patients relapse despite MRD negativity, why some MRD-positive states remain indolent, why response durability differs after immunotherapy, and why infection risk and immune attrition may limit the benefit of continuous T-cell redirection. The most productive direction is not to create parallel MRD and immune risk systems, but to combine them into a single monitoring framework that captures residual tumor burden and residual immune control (28, 36).

From a clinical perspective, the strongest immediate applications are in trial design and risk-adapted surveillance. MRD positivity can enrich for patients who need intensification studies, while sustained MRD negativity can identify candidates for de-escalation or treatment discontinuation trials. Immune profiling can refine both groups by identifying patients whose biology is discordant with their MRD result. Over time, prospective validation may support immune-informed MRD decisions in routine care (15, 66).

## 13 Conclusion

The management of multiple myeloma is entering an era in which prognosis is increasingly defined over time rather than only at diagnosis. MRD assessment has transformed response evaluation and remains the most validated dynamic biomarker of disease burden. Immune profiling provides the complementary dimension required to understand whether residual disease is likely to remain controlled or re-emerge. Integrating MRD kinetics, immune reconstitution, imaging, liquid biopsy, and computational modeling may allow a more precise, response-adapted approach to treatment intensity and duration (67). At present, however, integrated MRD–immune monitoring should be regarded primarily as a framework for biological interpretation, risk-adapted surveillance, and prospective clinical trial design, rather than as a validated basis for routine treatment modification. At present, however, integrated MRD–immune monitoring should be regarded primarily as a framework for biological interpretation, risk-adapted surveillance, and the design of prospective clinical trials, rather than as a validated basis for routine treatment modification. The central challenge for the next generation of trials is to convert this biological insight into validated therapeutic algorithms that improve survival, reduce toxicity, and move selected patients toward durable treatment-free remission.
